# Photocatalytic Performance and Degradation Pathway of Rhodamine B with TS-1/C_3_N_4_ Composite under Visible Light

**DOI:** 10.3390/nano10040756

**Published:** 2020-04-15

**Authors:** Jingjing Yang, Hongqing Zhu, Yuan Peng, Pengxi Li, Shuyan Chen, Bing Yang, Jinzhong Zhang

**Affiliations:** 1 Department of Environment and Quality Test, Chongqing Chemical Industry Vocational College, Chongqing 401228, China; hahajing1229@163.com (J.Y.); lipengxi616@163.com (P.L.); sychentsinghua@126.com (S.C.); 2 College of Resources and Environment, Southwest University, Chongqing 400715, China; hongqzhu@yeah.net; 3 College of Materials Science and Engineering, Yangtze Normal University, Chongqing 408100, China; ypeng@yznu.edu.cn

**Keywords:** TS-1 zeolite, C_3_N_4_, photocatalytic performance, Rhodamine B, degradation pathways

## Abstract

TS-1/C_3_N_4_ composites were prepared by calcining the precursors with cooling crystallization method and were characterized by scanning electron microscopy (SEM), transmission electron microscopy (TEM), Fourier transform infrared spectroscopy (FTIR), X-ray diffraction analysis (XRD), UV-Vis diffuse reflection spectrum (DRS) and nitrogen adsorption–desorption isotherm. The photocatalytic performance of TS-1/C_3_N_4_ composites was investigated to degrade Rhodamine B (RhB) under visible light irradiation. The results showed that all composites exhibited better photocatalytic performance than pristine TS-1 and C_3_N_4_; TS-1/C_3_N_4_-B composite (the measured mass ratio of TS-1 to C_3_N_4_ is 1:4) had best performance, with a rate constant of 0.04166 min^−1^, which is about two and ten times higher than those of C_3_N_4_ and TS-1, respectively. We attributed the enhanced photocatalytic performance of TC-B to the optimized heterostructure formed by TS-1 and C_3_N_4_ with proper proportion. From the results of photoluminescence spectra (PL) and the enhanced photocurrent, it is concluded that photogenerated electrons and holes were separated more effectively in TS-1/C_3_N_4_ composites. The contribution of the three main active species for photocatalytic degradation followed a decreasing order of ·O_2_^−^, ·OH and *h*^+^. The degradation products of RhB were identified by liquid chromatography tandem mass spectrometry (LC-MS/MS), and the possible photocatalytic degradation pathways were proposed.

## 1. Introduction

Photocatalysis is considered as a decisive way to cope with energy crises and solve environmental problems. Since the photocatalytic phenomenon of TiO_2_ was discovered by Honda and Fujishima in 1972 [1], the studies of photocatalysis have expanded, especially in the 21st century, to include photocatalytic water splitting [2,3,4], photocatalytic degradation of organic pollutants [5,6], photocatalytic reduction of CO_2_ [7,8] and photocatalytic organic synthesis [9,10]. Meanwhile, many semiconductor photocatalysts have been developed, and some traditional industrial catalysts have also been used as the photocatalysts. 

Conventionally, TS-1 zeolite (TS-1) has been used as an industrial catalyst to promote the selective oxidation of some organic compounds, such as the hydroxylation of aromatic hydrocarbons [11], the ammoxidation of ketones [12,13], the epoxidation of olefins [14], and the oxidation of saturated alkanes [15]. Recently, TS-1 has been confirmed as a promising photocatalyst in degradation of organic dye contaminants and selective conversion of propylene [16,17,18]. Due to its wide band-gap [19], TS-1 only responds to ultraviolet light. However, ultraviolet light accounts for only 5% of sunlight, while visible light accounts for 43%; thus, the application of TS-1 as a photocatalyst is limited. 

In order to utilize visible light, some efforts have been made to combine Fenton reaction and TS-1 photocatalysis in the activation of H_2_O_2_ [20,21,22]. A heterogeneous catalyst (Fe/TS-1 zeolite) and its application in the decoloration of Acid Orange 7 were reported [20]. Lv et al. [21] studied the photocatalytic activity in the degradation of phenol by using *γ*-Fe_2_O_3_/TS-1 with core–shell structure, and stated that the core *γ*-Fe_2_O_3_ served as a Fenton-like catalyst. Similar to photo-Fenton catalysis, the selective oxidation of benzene to phenol was investigated by using Fe-CN/TS-1 as photocatalyst under visible light, and the effects of Fe and H_2_O_2_ on photocatalytic performance were also examined [22].

TS-1 has also been used to broaden the application of sunlight by coupling with another photocatalyst that responds to visible light. Adepu et al. [23] pointed out that TS-1/BiVO_4_ displayed better photocatalytic Rhodamine B (RhB) degradation performance under sunlight irradiation. Photocatalytic ability of TS-1 could be enhanced by coupling with BiVO_4_, similar to some other semiconductor photocatalysts [24]. TS-1 has high specific surface area [25] and is very conducive to the adsorption of organic pollutants. Therefore, TS-1 is promising to be used as a photocatalyst in the field of organic wastewater treatment through combining visible-light-responsive photocatalysts to build hybrid system.

Among the visible-light-responsive photocatalysts, graphitic-C_3_N_4_ with a band gap of 2.7 eV has attracted special attention since it was discovered by Wang et al. [26] because of its advantages of response to visible light, environmental friendliness and stable chemical and thermal properties. Although C_3_N_4_ presents considerable photocatalytic performance, it is limited by its high recombination of photoexcited electron–hole pairs and low specific surface area. Many strategies have been employed to enhance its photocatalytic activity, such as doping nonmetal elements (B, S, F) [27,28,29], loading graphene and noble metal Pt [30,31] or transition-metal oxide (NiO) [32], combining other semiconductor photocatalysts (SiC [33], CdS [34], TiO_2_ [35]) and fabricating mesoporous C_3_N_4_ [36]. Due to the high specific surface area of TS-1 and good photocatalytic performance of C_3_N_4_, it is interesting to investigate the photocatalytic performance of TS-1/C_3_N_4_ composites under visible light.

In this work, the composites of TS-1 zeolite and C_3_N_4_ were prepared by calcining TS-1 and melamine with cooling crystallization method, and the photocatalytic degradation of Rhodamine B (RhB) was investigated with the prepared composites under visible light. Although RhB has been widely studied as a usual pollutant, its photocatalytic degradation pathways and degradation intermediates are not settled and have yet to be explored. 

## 2. Materials and Methods

### 2.1. Materials and Reagents

All chemical reagents were provided by China National Pharmaceutical Group Co., Ltd (Beijing, China). TS-1 zeolite (size: 250 nm, molar ratio of Si/Ti = 30) was provided by Sinopec Research Institute of Petroleum Processing, Beijing, China. Deionized water was used in all the experiments. 

### 2.2. Preparation of TS-1/C_3_N_4_ Composites

Firstly, triplet melamine (1.2 g) was dissolved well in 60 mL of deionized water at 90 °C, and a certain amount of TS-1 zeolite (0.05, 0.02 and 0.01 g) was added and dispersed by ultrasound for 10 min. Then, the mixture was stirred magnetically at 90 °C for 10 min. The ultrasonic and stirring processes were repeated three times. Afterwards, the mixture solution was cooled down to room temperature (about 20 °C), and 1 g melamine was precipitated due to the reduction of melamine solubility [37] (The solubility of melamine is about 0.33 g in 100 mL of water at 20 °C, so there is still 0.2 g of melamine dissolved in 60 mL of water). The resulting solid containing melamine and TS-1 zeolite was filtered with 0.45-μm filter membrane and dried at 80 °C for 1 h. Then, it was calcined in a muffle furnace at 550 °C for 2 h with a heating rate of 5 °C min^−1^ from room temperature to obtain the final products, which are denoted as TC-A, TC-B and TC-C (T refers to TS-1; C refers to C_3_N_4_; A,B,C refers to the added amount of TS-1 zeolite is 0.05 g, 0.02 g and 0.01 g during preparation of composites respectively).

### 2.3. Characterization of TS-1/C_3_N_4_ Composites

The prepared TS-1/C_3_N_4_ composites were characterized by powder X-ray diffraction (XRD, Rigaku SmartLab 3 kW, Tokyo, Japan). The morphologies of the composites were examined by field-emission scanning electron microscopy (FE-SEM, FEI Quanta 400 FEG, Hillsboro, OR, USA) and transmission electron microscopy (TEM, Philips Tacnai F20, Amsterdam, Holland). The UV-Vis diffuse reflectance spectra of the composites were recorded by UV-Vis spectrophotometer (Lambda 650, PerkinElmer, Waltham, MA, USA), in which BaSO_4_ was used as the reflectance standard. The Brunauer–Emmett–Teller (BET) surface areas of the composites were measured with nitrogen adsorption and desorption on a Micromeritics ASAP 2020 Physisorption (Atlanta, GA, USA). The pore size distribution of the composites was calculated by the Barrett–Joyner–Halenda method (BJH), and Fourier transform infrared spectra (FTIR) was performed using a PerkinElmer spectrometer (Waltham of MA, USA). Photoluminescence spectra (PL) of the composites were acquired on multifunctional vacuum ultraviolet fluorescence spectrometer (iHR320, Horiba, Japan). The mass ratios of C and N elements in TS-1/C_3_N_4_ composites were estimated by element analyzer (EA, Elementar Vario EL cube, Langenselbold, Germany).

### 2.4. Photocatalytic Test

#### 2.4.1. Photocatalytic Degradation of RhB

TS-1/C_3_N_4_ composite (0.01 g) was added into 100 mL of RhB solution (10 mg·L^−1^) and was stirred magnetically in the dark for 10 min; then, 0.5 mL of H_2_O_2_ was added. The mixture was irradiated by 300 W Xe arc lamp (PLS-SXE300, Beijing Trusttech Co. Ltd., Beijing, China) with a 420 nm cut-off filter positioned 5 cm away from the reactor. Irradiation took place at room temperature and ambient pressure under the condition of continuous stirring to keep the composite particles dispersed homogenously. The suspensions (4 mL) were taken out every 10 min and centrifuged at a rate of 13,000 rpm for 5 min. RhB concentration in the filtrate was measured at 554 nm by UV-Vis spectrophotometer (TU1901, Beijing Purkinje General Instrument Co., Ltd., Beijing, China), and the degradation products of RhB were identified by Waters Acquity UPLC-MS/MS (Milford, MA, USA). The total organic carbon (TOC) was measured on a TOC analyzer (HTY-CT1000M, Anatailin, Hangzhou, China).

#### 2.4.2. Photocatalytic Degradation of Dimethyl Sulfoxide (DMSO) 

TC-B composite (0.02 g) was added into 100 mL of DMSO solution (1 mg/mL). The other steps were exactly the same as in the degradation experiment of RhB. At certain intervals, 1 mL suspensions were withdrawn and filtered with 0.45-μm filter membrane to remove the photocatalyst. The concentration of DMSO was examined by high-performance liquid chromatography (HPLC) (Agilent Technologies 1200 series, Palo Alto, CA, USA) with C18 column at 224 nm. The mobile phase was acetonitrile and water (volume ratio: 6:94) with the flow rate of 1 mL/min. The elution time of DMSO was 1.646 min.

### 2.5. Photoelectrochemical Measurement

A photoelectrochemical test was conducted in a beaker with 0.01 g of the composite dispersed in 100 mL of 0.1 M Na_2_SO_4_ solution, using a CHI-660E electrochemical workstation (Chenhua Instruments Co., Shanghai, China) with saturated calomel electrode used as reference electrode and Pt electrode used as working electrode and counter electrode.

### 2.6. Trapping Experiments of Active Oxidized Species

In order to detect the active oxidized species during the photocatalytic process, 2-propanol (IPA) (0.25 mL), disodium ethylene diamine tetra acetic acid (EDTA-2Na) (0.18 g) and 1,4-benzoquinone (BQ) (0.028 g) were used as sacrificial agents to trap hydroxyl radicals (·OH), holes (*h*^+^) and superoxide radical anions (·O_2_^−^), respectively.

## 3. Results and Discussion

### 3.1. Characterization of TS-1/C_3_N_4_ Composites

**Figure 1:**
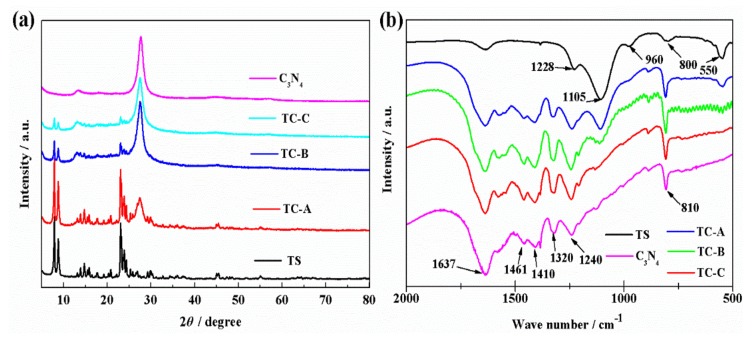
XRD patterns (**a**) and FTIR spectra (**b**) of all the materials.

The XRD patterns of all the materials are shown in Figure 1a. For C_3_N_4_, the peaks at 13.0 and 27.4° correspond to (100) and (002) planes of g-C_3_N_4_, respectively [38] (JCPDS Card No. 87–1526). The diffraction peaks of TS-1 and TS-1/C_3_N_4_ composites (8.2, 9.1, 23.4, 24.1 and 24.7°) belong to orthorhombic symmetry, corresponding to the MFI zeolitic structure [39]. The peaks of TS-1 tend to be weaker, whereas the peaks of C_3_N_4_ tend to be stronger, with a higher C_3_N_4_ content in TS-1/C_3_N_4_ composites. This is consistent with other heterostructures [40]. A similar phenomenon is also found in FTIR spectra of all materials, as shown in Figure 1b. The absorption peaks at frequencies of about 1637, 1461, 1410, 1320 and 1240 cm^−1^ are ascribed to the typical stretching vibration of aromatic C-N heterocycles in g-C_3_N_4_ [41]. The sharp absorption band at 810 cm^−1^ is caused by the C-N plane bending. Several bands located at about 550, 800, 1105 and 1228 cm^−1^ are attributed to the MFI zeolitic structure of TS-1 zeolite [19,42]. The peak at 960 cm^−1^ is assigned as characteristic of Ti framework [19], indicating the presence of Ti in tetrahedral coordination within the silicalite framework [43]. All the FTIR spectra of TC-A, TC-B and TC-C contain the bands of TS-1 and C_3_N_4_. The C and N element analysis data of TS-1/C_3_N_4_ composites are listed in Table 1. By evaluating the results of elemental analysis, the mass ratios of TS-1 to C_3_N_4_ are 1:2, 1:4 and 1:7 in TC-A, TC-B and TC-C, respectively, indicating that the TS-1/C_3_N_4_ composites were prepared successfully.

**Figure 2:**
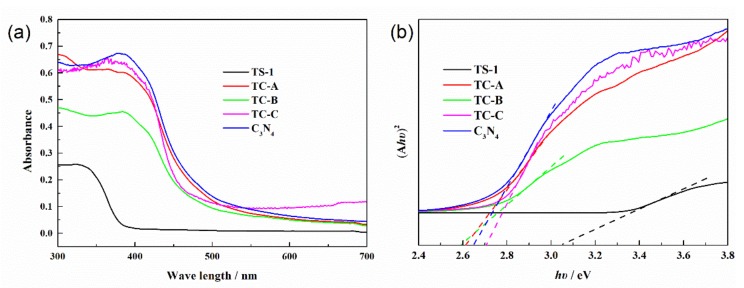
UV-Vis DRS spectra (**a**) and Tauc plots (**b**) of all the materials.

The UV-Vis diffuse reflectance absorption spectra and Tauc plots of the materials are shown in Figure 2. It can be seen from Figure 2a that the absorption edges of the composites are close to that of C_3_N_4_ due to the higher C_3_N_4_ content than TS-1 in composites. In Figure 2b, the indirect optical band gap can be obtained from the intercept of the resulting linear region with the energy axis at (*hν*)^2^ = 0 [44]. The band gap of C_3_N_4_ is 2.65 eV [45], which means that C_3_N_4_ can be excited to generate electrons and holes under visible light. Pristine TS-1 responds to UV light instead of visible light because its band gap is 3.05 eV. Therefore, TS-1 is barely excited to generate photo-electrons and photo-holes under visible light (*λ* ≥ 420 nm). The band gaps of TC-A and TC-B are lower than that of pristine C_3_N_4_. This indicates that the light utilization efficiency of TC-A and TC-B is improved. TS-1 is able to tune the band gap of the composites.

**Figure 3:**
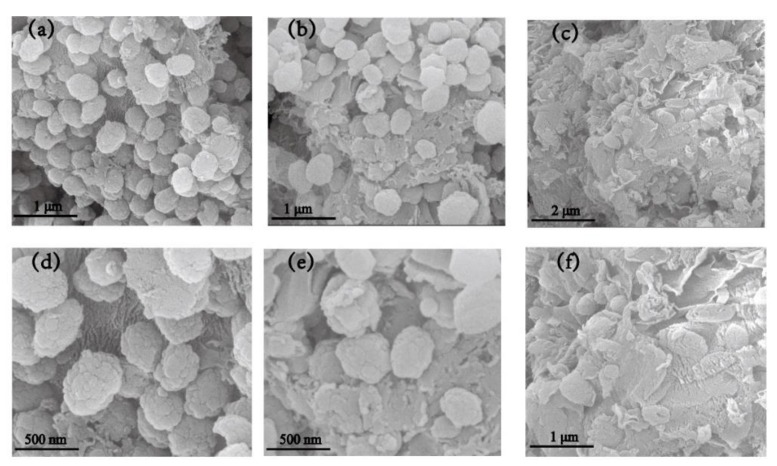
SEM images of TC-A (**a**,**d**), TC-B (**b**,**e**) and TC-C (**c**,**f**).

The SEM images of TS-1/C_3_N_4_ composites are shown in Figure 3. From Figure 3a, the average diameter of TS-1 sphere is 250 nm. For TC-A (Figure 3a,d), TS-1 spheres are deposited on the surface of block-structured C_3_N_4_. A bit of C_3_N_4_ is loaded on the surfaces of a small number of TS-1 spheres. With the increase of C_3_N_4_ content in TS-1/C_3_N_4_ composites, some TS-1 spheres in TC-B are deposited on the surfaces of the C_3_N_4_ blocks, while some are wrapped in C_3_N_4_ (Figure 3b,e). The TS-1 and C_3_N_4_ form effective contact. However, TS-1 spheres in TC-C (Figure 3c,f) are partly hidden and partly visible, and the majority are enveloped in C_3_N_4_ sheets, which may induce the invalid transfer of photogenerated electrons and holes of C_3_N_4_. Moreover, the adsorption capacity of TS-1 may be obscured because it is buried. Different integrated forms between TS-1 and C_3_N_4_ in TS-1/C_3_N_4_ composites may affect the transfer efficiency of photogenerated electrons and holes and further influence the photocatalytic degradation efficiency of RhB. Under visible light irradiation, the photogenerated electrons and holes of C_3_N_4_ in TC-A and TC-B may be effectively separated. This speculation needs be verified by photocatalytic degradation experiments.

**Figure 4:**
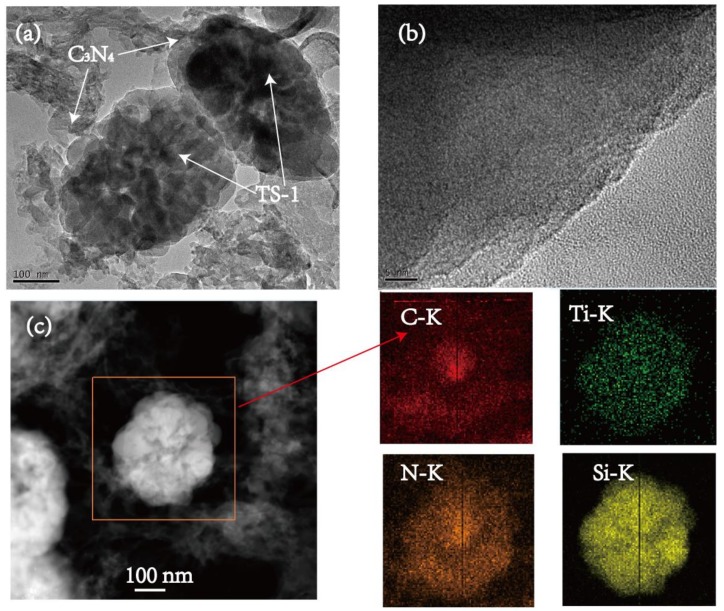
TEM (**a**) and HRTEM (**b**) images of TC-B. (**c**) Element mapping images of the boxed area are shown for C-K, N-K, Ti-K and Si-K.

The surface morphology of TS-1 in TC-B is depicted in detail by TEM images and the element mapping images (Figure 4). The crimp lamellar structure of C_3_N_4_ in the surface of a TS-1 sphere with a diameter of 250 nm (Figure 4a) can be seen, indicating that the C_3_N_4_ layer is thin and has no effect on the particle size of TS-1. TS-1 appears darker, and g-C_3_N_4_ appears in a lighter area with different thickness (Figure 4b). Meanwhile, the element mapping images of TC-B clearly show the distribution of C, N, Ti and Si elements (Figure 4c). C and N elements are distributed throughout the region, while Ti and Si elements are distributed in TS-1 core region. These results indicate that the surface of the TS-1 sphere is covered by C_3_N_4_ nanosheets.

**Figure 5:**
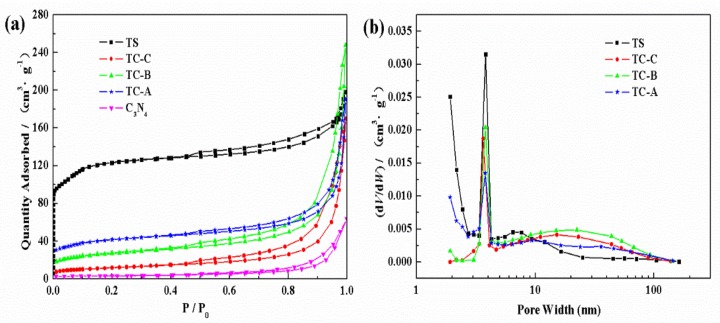
N_2_ adsorption–desorption isotherms (**a**) and the pore-widths of TS-1, C_3_N_4_, TC-A, TC-B and TC-C (**b**).

The N_2_ adsorption–desorption isotherms and pore sizes of the materials were evaluated by nitrogen adsorption–desorption measurement (Figure 5). It can be seen from Figure 5a that the adsorption–desorption isotherms correspond to the typical type IV isotherm [46,47], indicating that the composites are mesoporous. The BET specific surface area of C_3_N_4_ is 11 m^2^·g^−1^, which was investigated in our previous work [48]. The BET specific surface areas of TC-A, TC-B and TC-C are 138, 92 and 41 m^2^·g^−1^, respectively, which are all lower than that of 400 m^2^·g^−1^ for TS-1 zeolite. This phenomenon may be attributed to the fact that some TS-1 is covered by C_3_N_4_, so C_3_N_4_ may block some portion of the TS-1 pores. These results are in accordance with those of SEM and TEM. The average pore width of all the materials is about 5 nm (Figure 5b). Compared with TS-1, the pore widths of TC-A and TC-B do not change significantly, but the pore sizes of TC-C are slightly smaller, which may be due to the high content of C_3_N_4_ in TC-C and to more C_3_N_4_ entering the pores of TS-1. This phenomenon is different from the dispersion of C_3_N_4_ onto another Ti-contained mesoporous molecular sieve material, Ti-MCM-41. C_3_N_4_ enters the pores of Ti-MCM-41 and makes the pore size of Ti-MCM-41 increase due to partial collapse of the ordered hexagonal mesopore structure [49]. Different channel structure of TS-1 and Ti-MCM-41 may be the reason for this phenomenon. Regardless, due to its large BET surface area, uncovered TS-1 can adsorb RhB molecules to favor further degradation.

**Figure 6:**
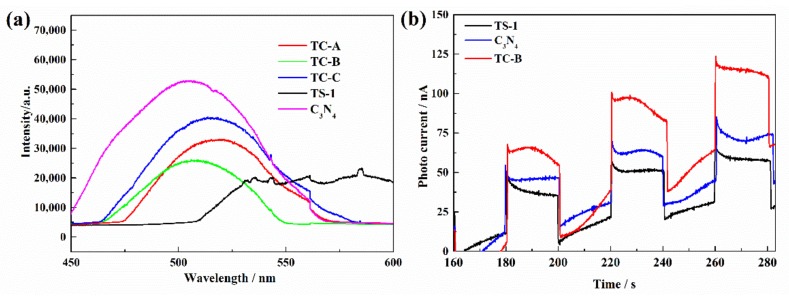
(**a**) PL spectra of all the materials. (**b**) The photocurrents of C_3_N_4_, TS and TC-B with light on/off cycles under visible light irradiation.

PL spectra were used to explore the electron–hole recombination of the materials (Figure 6a). It can be seen that C_3_N_4_ displays a higher emission peak, demonstrating that the electron–hole pairs are easily recombined. By contrast, TS-1/C_3_N_4_ composites display lower PL intensity, which means that the composites have lower electron–hole recombination. For these reasons, a better charge-carrier transfer under visible light irradiation is likely, and this can be confirmed by photocurrent response to light (Figure 6b). The photocurrent of TC-B is higher than those of TS-1 and C_3_N_4_, indicating the enhancement of separation efficiency of photo-induced electrons and holes in TC-B, which may be attributed to the synergetic effect between TS-1 and C_3_N_4_. The Ti in tetrahedral coordination within the silicalite framework may also be beneficial to the transfer of photogenerated electrons in g-C_3_N_4_ to TS-1, which is similar to the photogenerated electrons in g-C_3_N_4_ being transferred to the [Ti^4+^–O^2−^ ] active centers in Ti-MCM-41 [49].

### 3.2. Photocatalytic RhB Degradation Performance of TS-1/C_3_N_4_ Composites

**Figure 7:**
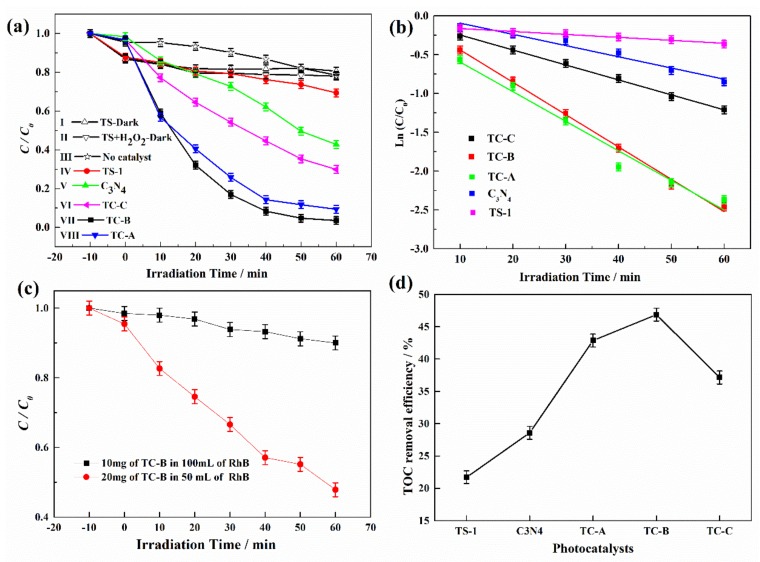
(**a**) Variations of RhB concentration with time. (**b**) The first-order kinetic fitting curves of RhB degradation with TS-1, C_3_N_4_, TC-A, TC-B and TC-C. (**c**) Time profiles of *C/C_0_* by TC-B in absence of H_2_O_2_ under different conditions. (**d**) Total organic carbon (TOC) removal efficiency of all photocatalysts.

The photocatalytic activities of the materials were evaluated by RhB degradation under visible light, and the results are shown in Figure 7. For comparison, photocatalytic activity of pristine TS-1 and C_3_N_4_ and the self-degradation of RhB without catalyst but in the presence of H_2_O_2_ were also evaluated (Figure 7a). *C/C*_0_ is used to describe the degradation performance, where *C* is the RhB concentration at time *t* and *C*_0_ is the initial concentration of RhB. Due to the large surface area of TS-1, the adsorption of TS-1 was also investigated. The presence or absence of H_2_O_2_ does not affect the adsorption capacity of TS-1 in dark reaction, and RhB concentration decreases by about 20% (curves I and II in Figure 7a). Although the specific surface areas of the materials vary greatly (Figure 5a), they show sufficient adsorption capacity of RhB before irradiation. Under visible light irradiation, RhB concentration decreases by only 20% within 60 min without catalyst (curve III in Figure 7a), which is attributed to the degradation in the presence of visible light and H_2_O_2_ [50]. H_2_O_2_ is a kind of reactant to produce the active species (e.g., ·OH) under irradiation, and ·OH can accelerate the oxidation of organic compounds [51]. When photocatalyst is absent, the production of ·OH is not sustainable. *C/C*_0_ is 70% with pristine TS-1, which means 30% degradation of RhB (curve IV in Figure 7a). Based on curves II and III, the decreased concentration in curve IV of Figure 7a is contributed to the combination of the adsorption of TS-1 and the actions of visible light and H_2_O_2_. No obvious dye- sensitizing effects were observed. RhB concentration decreases by 57% with C_3_N_4_ for 60 min irradiation (curve V in Figure 7a) and decreases by 91%, 97% and 70% with TC-A, TC-B and TC-C (curves VI–VIII in Figure 7a), respectively. These results indicate that TS-1/C_3_N_4_ composites display excellent photocatalytic performance compared to pristine TS-1 and C_3_N_4_. In TS-1/C_3_N_4_ composites, TS-1, as a semiconductor material, not only favors the dispersion of C_3_N_4_ but also is helpful in separating the photogenerated electrons and holes. Due to its high BET surface area, TS-1 can favor the adsorption of RhB molecules and make the degradation reaction more effective. In order to further study the effect of H_2_O_2_ on the reaction process, the concentrations of H_2_O_2_ before and after reaction were detected by UV-Vis spectra, and the results are shown in Figure S1. It is discovered that the concentration of H_2_O_2_ does not decrease after irradiated or non-irradiated reactions over TS-1 and H_2_O_2_. However, a slight increase was observed over TC-C composite and H_2_O_2_ under irradiation. The possible reason for this phenomenon is analyzed in the Supplementary Materials.

Figure 7b shows the time profiles of ln(*C/C_0_*), where the slope indicates the reaction rate constant and thus intuitively indicates the photocatalytic activity of the materials. It shows that RhB degradation occurs according to the first-order reaction kinetic model. The rate constants are 0.0038, 0.0145, 0.0381, 0.0417 and 0.0193 min^−^^1^ with TS-1, C_3_N_4_, TC-A, TC-B and TC-C, respectively. It is noteworthy that TC-B displays higher photocatalytic activity than the other four materials; its rate constant is about three and eleven times greater than those of C_3_N_4_ and TS-1, respectively. The rate constant of RhB degradation with TC-B is higher than that with TiO_2_/C_3_N_4_ materials [52,53].

The photocatalytic performance of TC-B in the absence of H_2_O_2_ is investigated, and the results are shown in Figure 7c. While increasing the dosage of photocatalyst per unit volume, the value of *C/C_0_* changes from 0.90 to 0.49. This means that the degradation efficiency reached 51%, starting from 10%. The catalyst TC-B is able to destroy RhB molecules with high dosage in absence of H_2_O_2_. The TOC removal efficiencies of all photocatalysts are shown in Figure 7d. The highest removal efficiency is 47% by TC-B in 60 min, which indicates that some RhB molecules were indeed degraded to CO_2_ and H_2_O, but there are still some degradation intermediates.

### 3.3. Photocatalytic Degradation Pathways of RhB

**Figure 8:**
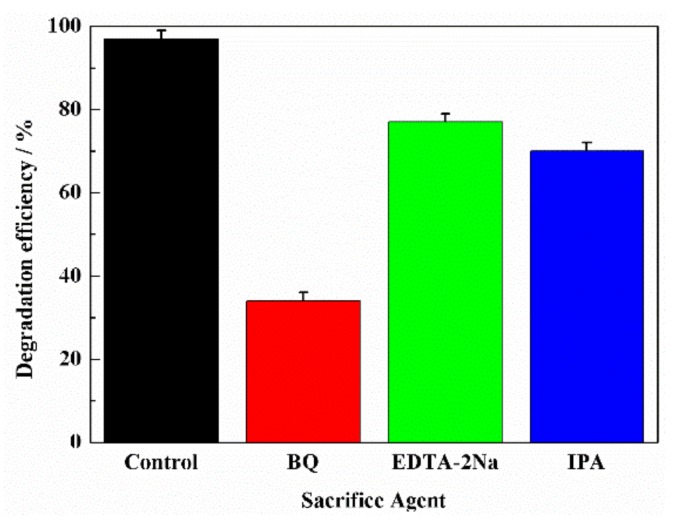
Trapping experiments of active species during the photocatalytic degradation of RhB with TC-B.

There are three oxidizing active species (·O_2_^−^, ·OH and *h*^+^) in the photocatalytic progress. ·O_2_^−^ could be generated by the conduction band electron-driven reduction of dissolved O_2_ in water. ·OH may come from reductive decomposition of H_2_O_2_ induced by visible light or photogenerated electrons. In order to investigate their roles, the trapping experiments of active species were conducted using different scavengers during the photocatalytic degradation processes of RhB with TC-B, and the results are shown in Figure 8. The degradation efficiencies of RhB decrease from 97% to 37%, 70% and 78% in the presence of BQ, IPA and EDTA-2Na for 60 min, respectively. The contribution of the three types of oxidizing active species follows a decreasing order of ·O_2_^−^, ·OH and *h*^+^, indicating that ·O_2_^−^ radicals are the main active species responsible for photodegradation of RhB.

**Figure 9:**
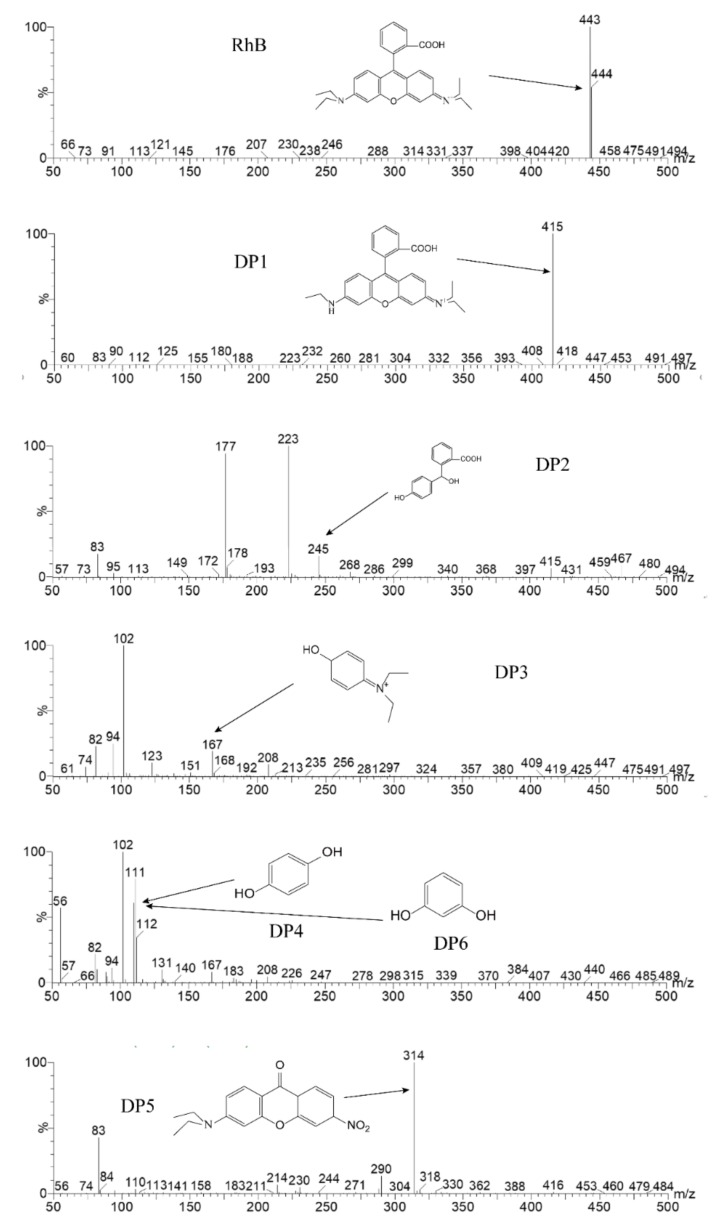
The identification results of the intermediates of RhB.

In order to gain insight into the degradation mechanism of RhB, LC-MS/MS was used to identify the intermediates generated in the degradation process. In contrast to previous studies [54,55], the reaction solutions were collected at 0, 10, 20, 30, 40, 50 and 60 min of degradation. The intermediates formed during degradation and the identification results are summarized in Table 2 and Figure 9. RhB concentration decreases with degradation time and cannot be detected after 50 min in this work. The intermediate with the *m*/*z* of 415 (DP1) exists before irradiation and reaches the maximum concentration at 30 min. Those with the *m*/*z* of 245 (DP2) and 167 (DP3) both appear at 20 min. Moreover, the intermediate DP2 cannot be detected at 30 min, but then DP3 concentration continuously increases and reaches the maximum at 50 min. In addition, the intermediate DP5 with the *m*/*z* of 314 appears at 10 min, and then its concentration continuously increases. The intermediate with the *m*/*z* of 111 (DP4 or DP6) appears at 20 min, and its concentration does not change.

**Figure 10:**
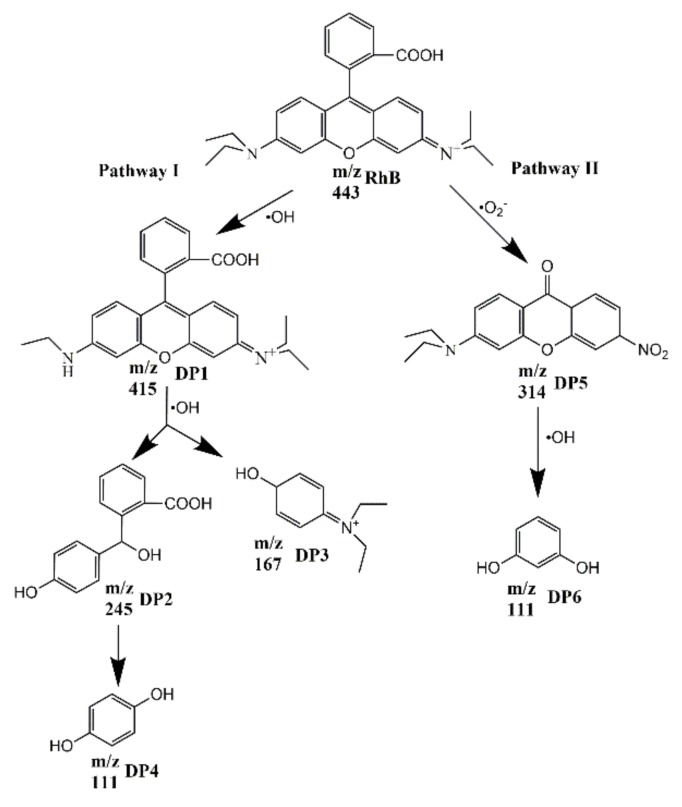
The possible photodegradation pathways of RhB.

Combined with the results of active species trapping experiments and LC-MS/MS, the photocatalytic degradation pathways are proposed in Figure 10. There are two possible degradation pathways. One is that RhB is attacked by the ·OH and de-ethylated to form DP1 with the *m*/*z* of 415 [54], which can be suggested as a de-ethylated product of RhB. Both direct photolysis of RhB and attack by the ·OH can make RhB form DP1 by elimination reactions. Under the continuous attack of ·OH, a double bond and tertiary amine in DP1 are broken, and DP1 is decomposed to DP2 and DP3, while DP2 is further decomposed to DP4. The other degradation pathway is that the positive amino group in the RhB molecule is attacked by ·O_2_^−^ and decomposed to DP5, and then DP5 is attacked by the ·OH to decompose to DP6. DP4 or DP6 may be further degraded to small molecular compounds. 

### 3.4. Extensive Applicability of TS-1/C_3_N_4_ Composites as Photocatalyst

**Figure 11:**
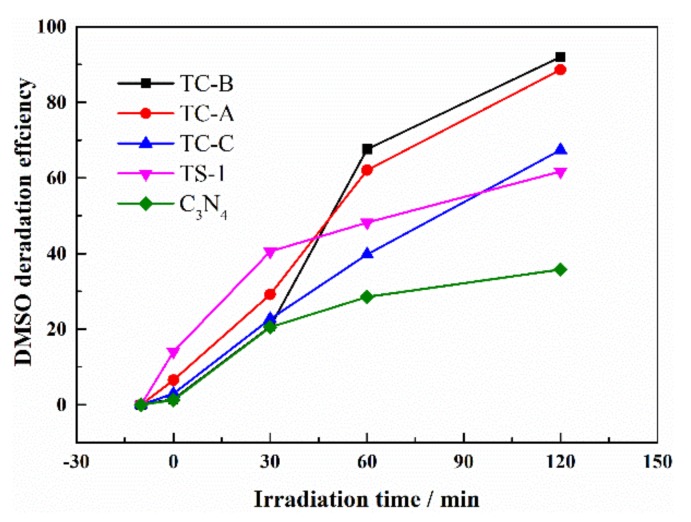
Time profiles of DMSO photocatalytic oxidation over all materials.

Rhodamine B, which we studied as a target pollutant, is a colored dye. In order to investigate the extensive applicability of the TS-1/C_3_N_4_ composite as a photocatalyst, the colorless organic molecule DMSO is used as the other target pollutant. It can be seen from Figure 11 that the photocatalytic DMSO efficiencies by TS-1, C_3_N_4_, TC-A, TC-B and TC-C are 62%, 36%, 67%, 88% and 92%, respectively, under visible light within 120 min. The TS-1/C_3_N_4_ composites show better performance than individual components do, and further mechanistic investigation is underway. It is proved that TS-1/C_3_N_4_ composites are worthy of extensive research as a potential photocatalyst.

## 4. Conclusions

TS-1/C_3_N_4_ composites have been successfully prepared and used to promote the photocatalytic degradation of RhB. The degradation efficiencies of RhB with TC-A, TC-B and TC-C reached 91%, 97% and 70%, respectively, and were higher than those with pristine TS-1 (30%) and C_3_N_4_ (57%). TS-1/C_3_N_4_ composites displayed lower PL intensity, and TC-B showed higher photocurrent than TS-1 and C_3_N_4_. The contact between TS-1 and C_3_N_4_ is favorable for the transfer of photogenerated electrons and holes. Synergistic effect of two components in TS-1/C_3_N_4_ and the effective separation of photo-generated charges are the key reasons for the improved photocatalytic efficiency. This work considered the degradation mechanism in detail. ·O_2_^−^ radicals are the main active species responsible for the photocatalytic degradation of RhB. The degradation intermediates of RhB were identified by LC-MS/MS, and two possible photocatalytic degradation pathways were discussed. This work demonstrated that TS-1/C_3_N_4_ could be used as a good photocatalyst in response to visible light.

## Figures and Tables

**Table 1:** The C and N elemental analysis data in composites.

Composites	Mass Percentage (%)		m(TS-1):m(C_3_N_4_)
	C	N	
TC-A	23.90	39.59	1:2
TC-B	30.10	49.79	1:4
TC-C	32.83	54.31	1:7

**Table 2:** RhB and intermediates identified by LC-MS/MS.

Irradiation Time (min)	*m*/*z*					
	443 (RhB)	415 (DP1)	314 (DP5)	245 (DP2)	167 (DP3)	111 (DP4/DP6)
0	◯ *	◯	/	/	/	/
10	◯	◯		/	/	/
20			◯	◯	◯	◯
30	◯	◯ *	◯	/	◯	◯
40	◯	◯	◯	/	◯	◯
50	/	/	◯	/	◯ *	◯
60	/	/	◯ *	/	◯	◯

◯: detected in LC; /: undetected in LC; *: reaches maximum concentration.

## Supplementary Materials

The following are available online at https://www.mdpi.com/2079-4991/10/4/756/s1, Figure S1: UV–vis adsorption spectral changes for the RhB solution in different conditions (**a**) TS-1+H_2_O_2_-unirradiate; (**b**) TS-1+H_2_O_2_-irradiate; (**c**) TC-C+H_2_O_2_-unirradiate, Table S1: A list of reactions that take place in different cases.
