# Supplementary material for: Photocatalytic Performance and Degradation Pathway of Rhodamine B with TS-1/C3N4 Composite under Visible Light
Source: Nanomaterials (Basel). 2020 Apr 15;10(4):756. doi: 10.3390/nano10040756 (PMC7221997; doi:10.3390/nano10040756)
Supplement: Supplementary file 1 [file nanomaterials-10-00756-s001.pdf]

Supplementary materials

Photocatalytic Performance and Degradation Pathway of Rhodamine B with TS-1/C_3N_4 Composite under Visible Light

Jingjing Yang [1], Hongqing Zhu [2], Yuan Peng [3], Pengxi Li [1], Shuyan Chen [1], Bing Yang [1,*] and Jinzhong Zhang [2,*]

1 Department of Environment and Quality Test, Chongqing Chemical Industry Vocational College, Chongqing 401228, China; hahajing1229@163.com (J.Y.); lipengxi616@163.com (P.L.); sychentsinghua@126.com (S.C.)

2 College of Resources and Environment, Southwest University, Chongqing 400715, China; hongqzhu@yeah.net

3 College of Materials Science and Engineering, Yangtze Normal University, Chongqing 408100, China; ypeng@yznu.edu.cn

* Correspondence: hyjxyangbing@163.com (B.Y.); jzhzhang@swu.edu.cn (J.Z.)

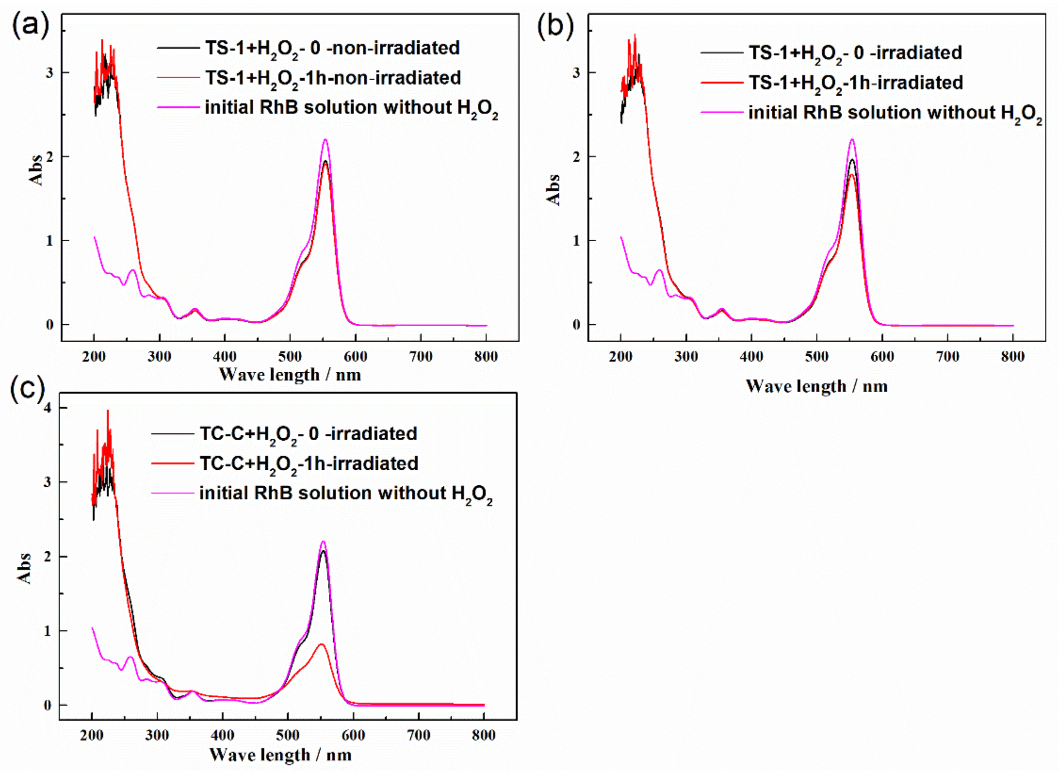

Figure S1. UV–vis adsorption spectral changes for the RhB solution in different conditions (**a**) TS-1+H_2O_2-unirradiate; (**b**) TS-1+H_2O_2-irradiate; (**c**) TC-C+H_2O_2-unirradiate.

In order to further study the effect of H_2O_2 on the reaction process during reaction, the concentration of H_2O_2 was detected by UV-Vis spectra and the results are shown in Figure S1. The absorption peak of is around 220 nm. It is discovered that the concentration of H_2O_2 does not decrease before and after reactions irradiated or non-irradiated over TS-1 and H_2O_2. But a little bit of an increase was observed over TC-C composite and H_2O_2 under irradiation.

The possible reasons for this phenomenon are proposed as below. C_3N_4 can generated e^- and h^+ under visible light, however the h^+ on valence band (VB) of C_3N_4 is too weak to oxide OH^- to $\cdot OH$. A series of reactions between the H_2O_2 and other oxidant species are listed as below [1–3].

$$\mathbf{H_2O_2 + h\upsilon \rightarrow \cdot OH} \quad (1)$$

$$\mathbf{H_2O_2 + Ti\text{-}OH \rightarrow Ti\text{-}O^{\cdot} + \cdot OH + H_2O} \quad (2)$$

$$\mathbf{H_2O_2 + e^- \rightarrow \cdot OH + OH^-} \quad (3)$$

$$\mathbf{O_2 + e^- \rightarrow \cdot O_2^-} \quad (4)$$

$$\mathbf{\cdot OH + \cdot OH \rightarrow H_2O_2} \quad (5)$$

$$\mathbf{H^+ + \cdot O_2^- \rightarrow HO_2^{\cdot}} \quad (6)$$

$$\mathbf{HO_2^{\cdot} + H^+ + e^- \rightarrow H_2O_2} \quad (7)$$

The H_2O_2 can be activated by visible light or e^- to decompose to $\cdot OH$ (reaction 1 and 3), and can react with Ti-OH to produce $\cdot OH$ (reaction 2). Dissolved O_2 in solution is reduced by e^- to form $\cdot O_2^-$ (reaction 4). Highly reactive hydroxyl radicals $\cdot OH$ can combine with each other to yield H_2O_2 (reaction 5). And H_2O_2 are also produced by reaction (6) and (7). In consequence, H_2O_2 is consumed in reactions (1) (2) (3) while supplemented by reactions (5) (6) (7). Visible light and active species (h^+ or $\cdot O_2^-$ or $\cdot OH$) can promote RhB degradation to some extent independently.The specific reactions take place in different cases are listed in Table S1.

Table S1 A list of reactions that take place in different cases.

case	reaction
TS-1+H_2O_2 non-irradiated	(2)(5)
TS-1+ H_2O_2 irradiated	(1)(2)(5)
TC-C+ H_2O_2 irradiated	(1)(2)(3)(4)(5)(6)(7)

For the first case, H_2O_2 can be activated by Ti-OH in TS-1 to produce $\cdot OH$. Some of $\cdot OH$ combined with each other to form H_2O_2, and the others react with RhB. From the results of photocatalysis (Figure 7), it is inferred that there is a very small amount of $\cdot OH$ to react with RhB, so the concentration of H_2O_2 changed little during dark reaction for one hour (Figure S1(a)). It is consistent with curves I and II in Figure 7(a). The presence or absence of H_2O_2 does not affect the degree of RhB concentration reduction (about 20%).

For the second case, TS-1 is not able to generate photo-electrons under visible light, so the circumstance is similar to the first one. It is concluded from Figure S1(b) that the decomposition rate of H_2O_2 is little (reaction (1)) under visible light. Compared curve II and IV (Figure7a), the more decreased concentration in curve IV than of curve II Figure 7a is contributed to the photolysis of visible light.

For the third case, the concentration of H_2O_2 has a little bit of an increase after irradiation for one hour (shown in Figure S1(c)). Although the large number of H_2O_2 involved in the reaction resulting in its consumption, it was supplemented by reactions (4), (5), (6). The phenomenon is consisted with the reference reported [2-3].

References:

[1] Zhong, W.; Qiao, T.; Dai, J.; Mao, L.; Xu, Q.; Zou, G.; Liu, X.; Yin, D.; Zhao, F. Visible-light-responsive sulfated vanadium-doped TS-1 with hollow structure: Enhanced photocatalytic activity in selective oxidation of cyclohexane. *J. Catal.* **2015**, *330*, 208–221.

[2] Cui, Y.; Ding, Z.; Liu, P.; Antonietti, M.; Fu, X.; Wang, X. Metal-free activation of H_2O_2 by g-C3N4 under visible light irradiation for the degradation of organic pollutants. *Phys. Chem. Chem. Phys.* **2012**, *14*, 1455–1462.

[3] Wang, L.; Cao, S.; Guo, K.; Wu, Z.; Ma, Z.; Piao, L. Simultaneous hydrogen and peroxide production by photocatalytic water splitting. *Chinese J. Catal.* **2019**, *40*, 470–475.
